# Increased health care utilization by survivors of childhood lymphoblastic leukemia is confined to those treated with cranial or total body irradiation: a case cohort study

**DOI:** 10.1186/1471-2407-14-419

**Published:** 2014-06-10

**Authors:** Anna Sällfors Holmqvist, Christian Moëll, Lars Hjorth, Anna Lindgren, Stanislaw Garwicz, Thomas Wiebe, Ingrid Øra

**Affiliations:** 1 Pediatric Oncology and Hematology, Skåne University Hospital, Clinical Sciences, Lund University, Lund, Sweden; 2 Center for Mathematical Sciences, Lund University, Lund, Sweden

**Keywords:** Childhood acute lymphoblastic leukemia, Survivors, Late complications, Morbidity, Health care utilization

## Abstract

**Background:**

Previous studies have indicated that survivors of childhood acute lymphoblastic leukemia (ALL) have an increased morbidity measured in terms of health care utilization. However, earlier studies have several potentially important limitations. To overcome some of these, we investigated hospital contact rates, and predictors thereof, among 5-year survivors of ALL in a population-based setting, and compared them to a control cohort regarding outcome measures from a comprehensive nation-wide health register.

**Methods:**

All individuals diagnosed with ALL before the age of 18 in Southern Sweden during 1970–1999 and alive January 2007 (n = 213; male = 107) were identified through the Swedish Cancer Register. Each subject was matched to fifty controls, identified in the Swedish Population Register. All study subjects were linked to the National Hospital Register and detailed information was obtained on all hospital contacts (hospital admissions and outpatients visits) starting five years after cancer diagnosis, and the corresponding date for the controls, until 2009.

**Results:**

The median follow-up among the 5-year survivors of ALL was 16 years (range 5–33), accruing a total of 3,527 person-years. Of the 213 5-year survivors, 105 (49.3%) had at least one hospital contact compared to 3,634 (34.1%) of the controls (p < 0.001). Survivors had more hospital contacts (3 [1–6] vs. 2 [1–4] contacts, p < 0.001) and more total days in hospital (6 [2–18] vs. 3 [1–7] days, p < 0.001) than the controls during the study period. Logistic regression analysis showed that survivors treated with cranial irradiation and/or total body irradiation (45% and 7%, respectively) had an increased risk of at least one hospital contact (OR 2.3, 95%CI; 1.5–3.6 and OR 11.0, 95%CI; 3.2–50.7, respectively), while there was no significant difference between the non-irradiated survivors and controls.

**Conclusions:**

We show that irradiated survivors of childhood ALL have an increased morbidity measured in terms of hospital contacts, in comparison to non-irradiated survivors and controls, while non-irradiated survivors have not. These findings are encouraging regarding the future morbidity of children currently treated for ALL, as radiotherapy is necessary only for a minority of these.

## Background

Survival rates for children with cancer have improved dramatically during the past four decades. Acute lymphoblastic leukemia (ALL), the most common childhood malignancy, accounts for approximately 25% of all pediatric cancers. Today, 85% of children with ALL treated in the Nordic countries survive more than five years [1].

As the population of adult survivors of childhood cancer grows, late complications, arising primarily from the cancer treatment, are becoming increasingly evident. These late effects include cardiovascular, endocrine, pulmonary, neurological and cognitive complications, as well as effects on fertility, musculoskeletal structures, hearing and vision. There is also an increased risk of secondary malignancies and premature death [2-10]. More than two thirds of survivors may develop a chronic condition as a long-term complication, and this fraction seems to increase with longer follow-up [11-13].

Health care utilization can be used as an indirect measure of overall morbidity. Two studies in British Columbia, Canada, comparing hospitalizations in a cohort of childhood cancer survivors with population controls, showed that survivors were hospitalized to a greater extent than the controls [14,15]. In the first study, where leukemia survivors were analyzed separately, an increased relative risk of hospitalization was seen, irrespective of treatment regime [15]. Self-reported hospitalization was found to be higher among survivors of leukemia in the North American Childhood Cancer Survivor Study (CCSS)[16]. In this article, the authors call for further research to fully describe health care utilization among childhood cancer survivors [16].

Health care utilization, as a measure of morbidity, is affected by the representativeness of the study group and the control group, as well as the collection of outcome data. Previous studies mostly report on health care utilization after all types of childhood cancer and not specifically on leukemia survivors. Other studies are limited by high rates of loss to follow-up, possible recall bias in questionnaire studies, and lack of appropriate control groups.

In-depth knowledge of the morbidity of childhood cancer survivors is essential for the further development of treatment protocols, improved prevention strategies, patient counseling and follow-up care. To clarify the extent and causes of hospital contacts among the large group of survivors of ALL in childhood, we conducted this population-based, retrospective study. The present study has the strength of including data from comprehensive nationwide registers, and a large matched control group from the general population, thereby avoiding some of the shortcomings of previous studies. We investigated the number of hospital contacts, the length of hospital stays and the diagnoses of the hospital contacts among ALL survivors and compared these to those of the control cohort. In addition, we investigated potential risk factors for morbidity, including relapse and treatment with cranial and total body irradiation, in order to facilitate specific health care planning for subgroups of ALL survivors in terms of screening for and management of chronic health conditions.

## Methods

### Study population

A database (BORISS, Childhood Cancer Register of Southern Sweden) has been established at the Department of Pediatric Oncology and Hematology, Skåne University Hospital, Lund, Sweden [17]. This database contains information on all individuals living in the Southern region of Sweden at the time of diagnosis (current population 1.7 million) who were diagnosed with cancer before the age of 18, during the period 1970–1999. BORISS was established in collaboration with the Swedish Cancer Register, which covers virtually all cases of cancer in Sweden since 1958 [18]. Information on diagnosis and treatment, collected from the medical records of 1,617 children diagnosed with cancer, are included in the BORISS database. In January 2007, 1,106 of these individuals were still alive, 213 (107 men, 106 women) of whom were survivors of ALL. None of the 213 survivors of ALL had Down’s syndrome, as confirmed by medical records.

### Control cohort

A randomly sampled control cohort of 10,650 individuals (50 controls per survivor) was identified in the Population Register of Sweden (Statistics Sweden), which contains basic demographic information on the entire Swedish population. The controls were matched for gender, year of birth and county of residence of the year of diagnosis of the corresponding patient. In Sweden, all individuals are assigned a unique personal identification number, which allows accurate linkage of information between various registers.

### Outcome variables

In the Swedish National Hospital Register, we identified all hospital admissions and outpatient visits (referred to as hospital contacts), as well as information on up to 20 discharge diagnoses for all hospital contacts from 5 years after the diagnosis of ALL, or the corresponding date for the controls, until the end of follow-up on December 31, 2009, for both cohorts. In the event of a relapse, hospital contacts within the first five years after diagnosis of relapse were excluded. The Swedish National Hospital Register includes information on hospital admissions in various parts of the country since 1964, and for the entire country since 1987; in addition, information on outpatient visits to hospitals is included for the entire country since 2001 [19]. Registration is mandatory. To reflect clinically meaningful entities of low, medium and high severity, the total number of hospital contacts was divided into three groups: 0, 1–2 or at least 3, and the sum of the number of days in hospital into four groups: 0, 1–7 days, 8–30 days or more than 30 days.

Discharge diagnoses during the study period are coded in the National Hospital Register according to three different versions of International Classification of Diseases (ICD); i.e., ICD-8 (1969–1986), ICD-9 (1987–1996) and ICD-10 (1997 and onwards). Hospital outpatient visits were included starting 2001. Discharge diagnoses related to pregnancy, delivery and perinatal period were excluded (ICD-10: O00–O99, ICD-9: 630–676, ICD-8: 630–678).

If the main discharge diagnosis recorded in the National Hospital Register was ALL (ICD-8: 204.0, 204.9, 207.0, ICD-9: 204, 207, 208, ICD-10: C910, C91.9, C950, C95.7, C95.9), the secondary diagnosis was chosen. If all discharge diagnoses (including the main diagnosis) registered for a specific hospital contact were ALL, it was excluded from the analysis since it most likely represented a routine follow-up after ALL, according to the standard in Sweden with follow-up of childhood cancer survivors at hospitals until at least the age of 18 years. On all other occasions, only the main discharge diagnosis was included in the analyses.

The main diagnostic groups were selected according to the ICD, except the group of infectious diseases, which in this study includes both the original main group entitled “Certain infectious and parasitic diseases” and diagnostic codes from other main groups including site-specific infections (meningitis, otitis, acute infections in the upper airways, influenza, pneumonia, other acute infections in the lower airways and infections of the airways).

Socio-economic data was collected from registers maintained by Statistics Sweden. The parents of the subjects were identified in the Swedish Multigenerational Register, which is part of the Population Register.

### Exposure variables

The information on cancer treatment collected from medical records includes type and amount of radiotherapy (continuous variable). The three ALL survivors who in addition to chemotherapy received both cranial irradiation and total body irradiation, were classified together with survivors treated with total body irradiation and chemotherapy. Information on possible relapse was collected from medical records. Age at diagnosis of ALL was divided into three groups; <5, 5–9 and ≥10 years of age. The highest level of education of the father and mother, respectively, was divided into five groups; less than 9 years, completed secondary school, high school, college/university and graduate school, respectively. The country of birth of the father was divided into three groups; Sweden, other Nordic country and other country. For the analyses of the influence of parents’ income, the annual income of the parent with the highest income at the year of diagnosis of the study subject, or the corresponding year of the control, was chosen.

The study was approved by the Regional Ethics Review Board, Lund University (official records numbers; 113/2007, 2010/98, 2010/504).

### Statistical analyses

Comparisons of the number of hospital contacts, the number of days in hospital and diagnoses of hospital contacts between survivors and controls were performed using the chi-squared test. The numbers of hospital contacts and days in hospital were also analyzed using the Mann–Whitney test. Comparisons regarding diagnosis of hospital contacts, as well as the socio-economic variables, between survivors and controls were performed using the chi-squared test. In these analyses p-values of <0.05 were considered significant. Continuous data are expressed as median and interquartile range unless stated otherwise.

The influence of treatment modalities and relapse on the risk of having at least one hospital contact was analyzed using multilevel logistic regression with each ALL survivor constituting a cluster together with its matched controls. The influence of treatment modalities and relapse on the number of hospital contacts and days in hospital was analyzed using ordinal logistic regression. No further adjustments were made. The impact of cranial irradiation dose on hospital contacts was investigated using logistic regression, adjusted for time period of diagnosis (decennium), gender, age at diagnosis, parent’s income, country of birth of father and highest level of education of the parents.

The influence of gender and age at diagnosis on the risk of having at least one hospital contact was analyzed using multilevel logistic regression, while the influence on the number of hospital contacts and days in hospital was analyzed using ordinal logistic regression. Interaction terms between these outcome variables and survivors were used and backward elimination of unnecessary factors according to Bayesian Information Criterion was used.

The statistical analyses were performed using the software package SPSS Statistics 16.0 and R version 3.0.1.

## Results

### Clinical characteristics

The clinical characteristics of the 213 survivors of ALL are presented in Table 1. Median age at diagnosis was 4 years (range 0–17) and 52% of the survivors were diagnosed with ALL before the age of five. The median follow-up among the 5-year survivors of ALL was 16 years (range 5–33) accruing a total of 3,527 person-years of follow-up. The control cohort constituted 10,650 individuals, monitored for a total of 176,750 person-years.

**Table 1:** Clinical characteristics of 5-year survivors of acute lymphoblastic leukemia

	**ALL survivors**		**Chemotherapy only**		**CRT**		**TBI**	
	**n = 213**	**%**	**n = 104**	**%**	**n = 95**	**%**	**n = 14**	**%**
Age at diagnosis								
<5 yrs	111	52.1	61	58.7	45	47.4	5	35.7
5-9 yrs	71	33.3	34	32.7	33	34.7	4	28.6
10-17 yrs	31	14.6	9	8.7	17	17.9	5	35.7
Period of diagnosis								
1970-1979	42	19.7	3	2.9	39	41.1	0	0
1980-1989	78	36.6	39	37.5	32	33.7	7	50.0
1990-1999	93	43.7	62	59.6	24	25.3	7	50.0
Age at end of follow-up								
10-19 yrs	54	25.4	46	44.2	6	6.3	2	14.3
20-29 yrs	71	33.3	39	37.5	26	27.4	6	42.9
30-39 yrs	73	34.3	15	14.4	53	55.8	5	35.7
>40 yrs	15	7.6	4	3.8	10	10.5	1	7.1
Relapse	24	11.3	2	1.9	11	11.6	11	78.6
Stem cell transplantation	14	6.6	0	0	0	0	14	100.0
Cranial irradiation								
12-15 Gy	2	0.9			2	2.1	0	0
18 Gy	28	13.1			28	28.4	1	7.1
20-23 Gy	6	2.8			6	6.3	0	0
24-30 Gy	62	29.1			60	63.2	2	14.3
Total body irradiation								
8-12 Gy	14	6.6					14	100.0

*Abbreviations:ALL* acute lymphoblastic leukemia, *CRT* received cranial irradiation in addition to chemotherapy, *TBI* received total body irradiation in addition to chemotherapy, including three cases who, in addition, received cranial irradiation.

The socio-economic characteristics of the ALL survivors and controls are presented in Table 2.

**Table 2:** Socio-economic characteristics concerning 5-year survivors of acute lymphoblastic leukemia and controls

	**ALL survivors**		**Controls**		**p-value**
	**n = 213**	**%**	**n = 10,650**	**%**	
Male	107	50.2	5350	50.2	1.0
Female	106	49.8	5300	49.8	
Born					
1965-1969	15	7.0	750	7.0	1.0
1970-1979	73	34.3	3650	34.2	
1980-1989	71	33.3	3550	33.3	
1990-1999	54	25.4	2700	25.4	
Highest level of education of father					
Less than 9 years	30	14.1	1545	14.5	0.943
Completed secondary school (9 years)	37	17.3	1636	15.3	
Graduated from high school	77	36.1	3857	36.2	
College/University	46	21.6	2227	20.9	
Graduate school	4	1.9	123	1.2	
Missing	19	8.9	1262	11.8	
Highest level of education of mother					
Less than 9 years	15	7.0	1021	9.6	0.771
Completed secondary school (9 years)	45	21.1	1845	17.3	
Graduated from high school	87	40.8	4340	40.8	
College/University	52	24.4	2523	23.7	
Graduate school	0	0	19	0.2	
Missing	14	6.6	902	8.5	
Country of birth of father					
Sweden	183	85.9	9055	85.0	0.324
Nordic country, except Sweden	10	4.7	315	3.0	
Other than Nordic country	19	8.9	1058	9.9	
Missing	1	0.5	222	2.1	

### Rate of hospital contacts

Of the 213 5-year survivors of ALL, 105 (49.3%) were admitted to hospital at least once during the study period starting five years after ALL diagnosis and ending in 2009, as were 3,634 (34.1%) of the controls (p < 0.001). In addition, among individuals with at least one admission, ALL survivors had more hospital contacts (in total 3 [1–6] vs. 2 [1–4], p < 0.001), and spent more days in hospital (in total 6 [2–18] vs. 3 [1–7] days, p < 0.001) than the controls during the study period (Table 3).

**Table 3:** Distribution (percentage) among 5-year survivors of acute lymphoblastic leukemia and controls by number of hospital contacts and days in hospital

	**Chemotherapy only**	**CRT**	**TBI**	**Relapse**	**ALL survivors**	**Controls**	**p-value**^**2**^
	**n = 104**	**n = 95**	**n = 14**	**n = 24**	**n = 213**	**n = 10,650**	
At least one hospital contact^1^	35.9	59.4	78.6	66.7	49.3	34.1	< 0.001
Total number of hospital contacts							
1–2	25.2	24.0	50.0	33.3	28.2	26.6	< 0.001
≥3	10.7	35.4	28.6	33.3	21.1	7.6	
Total number of days in hospital							
1–7 days	24.3	26.0	50.0	25.0	26.8	25.7	< 0.001
8–30 days	7.8	18.8	14.3	12.5	13.1	5.9	
> 30 days	3.9	14.6	14.3	29.2	9.4	2.5	

^1^Hospital contacts from 5 years after the diagnosis of ALL, or the corresponding date for the controls, until the end of follow-up. In the case of a relapse, hospital contacts within the first five yeas after diagnosis of relapse were excluded, ^2^p-value for comparison between all survivors and controls. *Abbreviations:CRT* received cranial irradiation in addition to chemotherapy, *TBI* received total body irradiation in addition to chemotherapy, including three cases who, in addition, received cranial irradiation.

### Diagnoses of hospital contact

Among those who were hospitalized at least once, diagnoses of infectious disease, benign neoplasms, diseases of the blood, endocrine diseases, diseases of the nervous system, eye, ear, circulatory system, and the skin were significantly more frequent in survivors than controls, as can be seen in Table 4. In contrast, the diagnosis of pulmonary disease was significantly more frequent among controls.

**Table 4:** Main diagnosis of hospital contact of survivors of acute lymphoblastic leukemia and controls

**Main diagnostic groups (ICD-8, ICD-9, ICD-10)**	**ALL survivors**		**Controls**		**p-value**^**2**^
	**n**	**%**	**n**	**%**	
Certain infectious and parasitic diseases (000–136, 001–139, A00-B99)^1^	20	9.4	475	4.5	0.002
Benign and *in situ* neoplasms and neoplasms of uncertain or unknown behavior (210–239, 210–239, D00-D48)	8	3.8	77	0.7	<0.001
Malignant neoplasm (140–209, 140–208, C00-C97)	1	0.5	3	0.0	NS
Diseases of the blood and blood-forming organs and certain disorders involving the immune mechanism (280–289, 280–289, D50-D89)	11	5.2	120	1.1	<0.001
Endocrine, nutritional and metabolic diseases (240–279, 240–279, E00-E90)	15	7.0	197	1.8	<0.001
Mental and behavioral disorders (290–315, 290–319, F00-F99)	9	4.2	380	3.6	NS
Diseases of the nervous system (320–358, 320–359, G00-G99; infections excluded)	11	5.2	130	1.2	<0.001
Diseases of the eye and adnexa (360–379, 360–379, H00-H59; infections excluded)	6	2.8	123	1.2	0.041
Diseases of the ear and mastoid process (380–389, 380–389, H60-H95; infections excluded)	6	2.8	128	1.2	0.048
Diseases of the circulatory system (390–458, 390–459, I00-I99; infections excluded)	7	3.3	152	1.4	0.037
Diseases of the respiratory system (460–519, 460–519, J00-J99; infections excluded)	2	0.9	434	4.1	0.013
Diseases of the digestive system (520–577, 520–579, K00-K93; infections excluded)	14	6.6	721	6.8	NS
Diseases of the skin and subcutaneous tissue (680–709, 680–709, L00-L99; infections excluded)	13	6.1	252	2.4	0.002
Diseases of the musculoskeletal system and connective tissue (710–738, 710–739, M00-M99; infections excluded)	5	2.3	335	3.1	NS
Diseases of the genitourinary system (580–629, 580–629, N00-N99; infections excluded)	18	8.5	586	5.5	NS
Injury, poisoning and certain other consequences of external causes (800–999, 800–999, S00-T98)	23	10.8	1,268	11.9	NS

^1^Including diagnostic codes from other main groups including site-specific infections (meningitis, otitis, acute infections in the upper airways, influenza, pneumonia, other acute infections in the lower airways, and infections of the airways, i.e. main groups VI-XIV. ^2^Chi^2^-test comparing survivors and controls.

### Treatment as a risk factor for hospital contacts

As can be seen from Table 1, 95 of the 213 survivors underwent cranial irradiation in addition to chemotherapy, and 14 underwent total body irradiation in addition to chemotherapy (including three cases in whom cranial radiation was given in addition to total body irradiation). Multi-level logistic regression analysis showed that survivors treated with cranial irradiation and/or total body irradiation, in addition to chemotherapy, had a significantly increased risk of having at least one hospital contact during the study period, compared to non-irradiated survivors and controls (OR 2.3 and 11.0, respectively) (Table 5). Among the subjects who had at least one hospital contact, survivors treated with cranial irradiation and/or total body irradiation had significantly more hospital contacts, with 3.9 and 4.8 times higher risks, than the non-irradiated survivors and controls (Table 5). Likewise, the number of days in hospital was increased only among those treated with cranial irradiation and/or total body irradiation compared to non-irradiated survivors and controls (OR 3.7 and 5.6, respectively) (Table 5).

**Table 5:** Odds ratios for hospital contacts of survivors of acute lymphoblastic leukemia compared to controls, according to treatment modalities

	**OR**	**95% CI**	**p-value**
**At least one hospital contact**^**1**^			
Controls	1		
Chemotherapy only	1.4	0.9–2.2	0.10
Cranial irradiation^2^	2.3	1.5–3.6	< 0.001
Total body irradiation^3^	11.0	3.2–50.7	< 0.001
**Increased number of hospital contacts**^**1**^			
Controls	1		
Chemotherapy only	1.1	0.8–1.7	0.53
Cranial irradiation^2^	3.9	2.6–5.9	< 0.001
Total body irradiation^3^	4.8	1.9–12.3	< 0.001
**Increased number of days in hospital**^**1**^			
Controls	1		
Chemotherapy only	1.2	0.8–1.7	0.43
Cranial irradiation^2^	3.7	2.5–5.5	< 0.001
Total body irradiation^3^	5.6	2.2–14.3	< 0.001

^1^Hospital contacts from 5 years after the diagnosis of ALL, or the corresponding date for the controls, until the end of follow-up. In the case of a relapse, hospital contacts within the first five yeas after diagnosis of relapse were excluded. ^2^Treated with cranial irradiation in addition to chemotherapy. ^3^Treated with total body irradiation in addition to chemotherapy. OR = odds ratio, CI = confidence interval.

There was no significant difference between the non-irradiated survivors and controls regarding the risk of being hospitalized at least once, the number of days in hospital or the number of hospital contacts (Table 5). The amount of cranial irradiation influenced the risk of hospital contacts. For every additional Gray of irradiation administered to the head, the risk of having at least one hospital contact increased by 5% (OR 1.05, 95% CI; 1.03–1.07).

Relapse did not alter the risk of hospital contacts, number of days in hospital, or number of hospital contacts when hospital contacts within the first five years after diagnosis of relapse were excluded, but the power for detecting a possible increase in risk is small due to the low number of relapses in the present study (Table 3).

### Socio-demographic risk factors for hospital contacts

Females more frequently had at least one hospital contact, and an increased number of hospital contacts and days in hospital than males, but this gender difference was the same among survivors and controls (Table 6). Females had at least one hospital contact to a significantly greater extent than males also when the subgroups of non-irradiated and irradiated survivors were analyzed and the interaction term between gender and treatment modality was non significant (data not shown). Age at diagnosis did not influence the risk of having at least one hospital contact. However, among patients with at least one hospital contact, age ≥ 10 years at diagnosis was associated with fewer hospital contacts and days in hospital (Table 6). The influence of socio-economic parameters did not differ between the group of survivors and the control cohort (data not shown).

**Table 6:** Odds ratios for hospital contacts by clinical characteristics

	**At least one hospital contact**			**Increased number of hospital contacts**			**Increased number of days in hospital**		
	**OR**	**95% CI**	**p-value**	**OR**	**95% CI**	**p-value**	**OR**	**95% CI**	**p-value**
**Study cohorts**									
Controls	reference								
ALL survivors	2.5	1.5–4.3	<0.001	2.7	1.6–4.3	<0.001	3.0	1.8–4.8	<0.001
**Gender**									
Male	reference								
Female	1.3	1.1–1.7	0.007	1.3	1.3–1.5	<0.001	1.3	1.2–1.4	<0.001
**Age at diagnosis**									
<5 yrs	reference								
5-9 yrs	1.0	0.8–1.3	0.94	1.0	0.9–1.1	0.72	1.0	0.9–1.1	0.73
10-17 yrs	0.9	0.6–1.2	0.40	0.9	0.8–1.0	0.02	0.9	0.8–1.0	0.03
**Interaction terms**									
Case-Female	0.8	0.5–1.5	0.55	0.9	0.5–1.6	0.76	0.9	0.5–1.5	0.56
Case-age 5–9 yrs at diagnosis	0.8	0.4–1.6	0.62	0.8	0.5–1.5	0.52	0.8	0.4–1.4	0.34
Case-age 10–17 yrs at diagnosis	0.5	0.2–1.2	0.15	0.5	0.2–1.1	0.10	0.4	0.2–1.0	0.05

OR = odds ratio, CI = confidence interval.

## Discussion

In this population-based study, we found that irradiated 5-year survivors of ALL had more hospital contacts and spent more days in hospital than both non-irradiated survivors and matched population controls. This risk increased with the amount of cranial irradiation received. In contrast, non-irradiated survivors of ALL did not have more hospital contacts, nor did they spend more days in hospital, than controls. Among those who were hospitalized at least once, a broad range of different diagnoses of the hospital contacts were significantly more frequent among ALL survivors than controls.

In the present study, comprising a follow-up from 1975 to 2009, 49% of survivors of ALL were hospitalized at least once, which is similar to the proportion found in leukemia survivors in British Columbia, Canada, where 40% had been hospitalized at least once (follow-up 1986–2000) [15]. Leukemia survivors included in the CCSS had a standardized incidence ratio of self-reported hospitalization of 1.4 (95%CI 1.3–1.4) compared with the general U.S. population [16]. In the BCCSS, 7.1 percent of survivors of leukemia reported having been hospitalized at least once during the observed year, a 1.4-fold risk compared with the general population (data from the National General Household Survey) [20]. Thus, the findings of the present study are also consistent with the results of the BCCSS and the CCSS. The BCCSS has a long period of follow-up and is, like the CCSS, a large study. However, both these studies rely on self-reported outcome measures. Furthermore, the CCSS includes patients only treated at the collaborating hospitals, while the present study is distinguished by being strictly population-based and includes outcome measures that are based on comprehensive nationwide registers. An additional strength of the present study is the use of a large, matched control cohort from the general population.

Our finding of a broad range of discharge diagnoses causing hospital contacts among survivors is supported in two comparable studies [15,16]. Clearly, the increased risk of hospital contacts among survivors of ALL compared to the controls is caused by many different diagnoses. One exception in our study is the finding of a smaller proportion of hospital contacts for pulmonary diseases in the group of ALL survivors than in the control cohort, which is in contrast to that of the two earlier studies. One may speculate that the different frequencies of pulmonary diseases in survivors and controls could be due to differences in smoking habits between cancer survivors and controls. This hypothesis is supported by the CCSS study, where survivors were found to smoke less than the general population [21]. Unfortunately, information on smoking was not available in the current study. We also found that survivors did not have hospital contacts to a greater extent for mental disorders, injuries or poisoning than controls, which is in contrast to previously published results [15,16].

It is well known that cranial and total body irradiation result in an increased risk of a broad range of late complications, and efforts to reduce radiotherapy in treatment protocols without compromising survival rates have been successful for ALL patients during the last three decades [22,23]. Nevertheless, Bradley et al. could not find a specific treatment modality to be a risk factor for hospitalization when survivors of all childhood cancers were grouped together [14]. When leukemia survivors were analyzed separately by Lorenzi et al., an increased risk of hospitalization was seen among survivors treated with chemotherapy only, as well as among those treated with both chemotherapy and cranial irradiation, compared to the control group [15]. In contrast to both these studies, we found an increased risk of having hospital contacts only in the subgroups of survivors whose treatment included radiotherapy in addition to chemotherapy. All three studies are population- and register-based, but the follow-up period of our study is longer, and we included survivors diagnosed with ALL as early as 1970, which may in part explain the disparities.

The main finding of our study that there is no increased risk of hospital contacts of non-irradiated survivors of ALL is supported by the CCSS study where the vast majority (92%) of the non-irradiated, non-relapsed survivors did not report any severe chronic medical condition [24]. On the basis of the results of the present study, follow-up of ALL survivors should primarily focus on developing preventive interventions, and enhancing patient counseling and follow-up care of those whose treatment included irradiation.

Our data show that, in both the survivor cohort and the control cohort, females had an increased risk for at least one hospital contact, more hospital contacts and longer hospital stays than males, which is in agreement with previous studies [15,16]. Moreover, we found that age at diagnosis did not influence the risk of having at least one hospital contact, but in the age group ≥ 10 years at cancer diagnosis, fewer hospital contacts and shorter hospital stays were seen. In the CCSS, young age at diagnosis was found to be associated with an increase in risk of hospitalization, while Lorenzi et al. showed that age at diagnosis did not significantly influence later hospitalization [15,16]. However, all types of childhood cancer were included in the analyses of the impact of gender and age at diagnosis in these two studies, making direct comparisons with our study difficult. In accordance with the study by Lorenzi et al., we found that the influence of socio-economic factors on hospital contacts did not differ between ALL survivors and controls.

The limitations of the current study must be considered. The Swedish National Hospital Register includes hospital-based outpatient data only since 2001, and no data from general practitioners are included, so the present study mainly captures conditions resulting in admission to hospital. In addition, the Swedish Hospital Register has national coverage only since 1987, which further reduces the number of hospital contacts included in the study. However, most parts of Southern Sweden have had complete coverage since 1970. The current study was not powered to look at the influence of treatment era on health care utilization. However, we did analyze the impact of treatment modality, which in part could be seen as a substitute for treatment era. Information on emigration and death was not considered in the follow-up of the control cohort, which could potentially have led to the underestimation of the rate of hospital contacts in this cohort. However, given the size of the control cohort, this potential underestimation should not be of any substantial significance.

## Conclusions

In conclusion, we have demonstrated that irradiated survivors of ALL have an increased morbidity expressed in terms of hospital contacts, compared to non-irradiated survivors and controls from the general population. In contrast, non-irradiated survivors of ALL did not have more frequent hospital contacts or more days in hospital than the controls. A broad range of different diagnoses of the hospital contacts were significantly more frequent in the ALL survivor cohort than in the control cohort. Survivors did, however, not have hospital contacts for mental diseases, injuries or poisoning, to a greater extent than the controls, and they had a lower frequency of hospital contacts due to pulmonary disease. This strictly population-based study, based on comprehensive nationwide registers including over 30 years of follow-up, is encouraging regarding morbidity for patients currently treated for ALL, as radiotherapy is considered necessary in only a minority.

## Abbreviations

ALL: Acute lymphoblastic leukemia; OR: Odds ratio; CI: Confidence interval; CCSS: the Childhood Cancer Survivor Study; BCCSS: the British Childhood Cancer Survivor Study; ICD: International Classification of Diseases.

## Competing interests

The authors declare that they have no conflicts of interest.

## Authors’ contributions

ASH, CM, LH, SG, TW and IØ contributed to the design of the study, and the collection of data. AL, ASH and IØ performed the data analyses. ASH and IØ drafted the manuscript. All authors made substantial contributions to the interpretation of the results. All authors contributed to editing and critical review of the manuscript regarding important intellectual content and all approved the final version.

## Pre-publication history

The pre-publication history for this paper can be accessed here:

http://www.biomedcentral.com/1471-2407/14/419/prepub
